# Reassessing the Role of σ Holes in Noncovalent Interactions: It is Pauli Repulsion that Counts

**DOI:** 10.3389/fchem.2022.858946

**Published:** 2022-04-07

**Authors:** Małgorzata M. Szczęśniak, Grzegorz Chałasinski

**Affiliations:** ^1^ Department of Chemistry, Oakland University, Rochester, MI, United States; ^2^ Faculty of Chemistry, Warsaw University, Warszawa, Poland

**Keywords:** sigma holes, Pauli repulsion, noncovalent interactions, donor–acceptor interactions, SAPT

## Abstract

A number of prototypical weak electron donor–electron acceptor complexes are investigated by the Symmetry Adapted Perturbation Theory, some of which belong to novel classes of weak bonds such as halogen and chalcogen bonds. Also included are complexes involving strong Lewis acids such as BeO and AuF. The common view in the literature is to associate these novel bonds with a variety of “holes”, *σ*, *π*, *δ*, or positive areas in their electrostatic potential maps. The presumption is that these positive areas of the electrostatic potential are indicative of the electrostatic nature of these noncovalent bonds. The electrostatic view extends to the explanations of the directionality of approaches between the subsystems forming these bonds. This work demonstrates that one common feature of these electrostatic potential “holes” is the local depletion of electron density of which the best detector is the first-order Pauli repulsion. The minimization of this repulsion determines the bond directionality and its relative angular rigidity. In relatively strong complexes of BeO with rare gases, where BeO shows a clear cavity in electron density—an ultimate “σ hole”—the electrostatic effect does not control the bending potential—the exchange repulsion does. In halogen bonds, the halogen atom is nonspherical, displaying an axial “σ hole” in its electrostatic potential. However, in no examined case, from rare gas acting as an electron donor to a polar donor to an anionic donor, is the electrostatic energy responsible for the directionality of the halogen bond. In fact, it is not even maximized in the direction of the σ hole in N_2_-ClF and NH_3_-ClF. Yet, in all the cases, the exchange repulsion is minimized in the direction of the σ hole. The minimized exchange repulsion associated with the subtle and less subtle depletions of the electron density occur on the nodal planes or on the intersections thereof in the highest occupied molecular orbitals of Lewis acids, provided that the systems are closed-shell. The role of nodal planes in covalent and coordinate covalent bonds is well recognized. This work points to their similarly equal importance in certain types of donor–acceptor noncovalent interactions.

## Introduction

The weak donor–acceptor interactions encompass a wide swath of noncovalent interactions from noble-gas molecule to halogen-bonded to metalophilic. New types are being discovered by examinations of crystal structures (Ramasubbu et al., 1986; Bauzá et al., 2013; Saha and Desiraju, 2017; Saha et al., 2018) while new names are added to the lexicons [for the latest list see (Zierkiewicz et al., 2021)]. One common feature of these interactions is the presence of broadly defined Lewis acid as an electron acceptor from an electron-rich Lewis base. The sources of electron deficiency may vary from the incomplete octet (as in BF_3_), low-lying LUMO [as in typical CT complex of Benesi and Hildebrand—benzene-I_2_ (Benesi and Hildebrand, 1948)], lower-lying T_2g_ ligand positions in O_h_ transition metal complexes, electron deficiency due to the positive quadrupole moment (as in dihalogens), to electropositive regions discussed by Legon (Legon, 1999), which morphed into ubiquitous “σ holes” (as in many types of novel bonds from halogen to tetrel) or actual cavities in electron distribution (as in BeO). In this work, we argue that these sources of electron deficiency have a common origin. For historical perspective, the terms σ hole initially referred to empty or half-empty σ-symmetry orbitals (Koch et al., 1987).

In today’s popular meaning of the role of “σ holes,” one refers to the map of electrostatic potential on a threshold density surface and to the locus of positive potential that is used to explain both the bonding and directionality of the interactions with a Lewis base. This explanation echoes the way that the positive partial charge on protons was used to explain the directionality of hydrogen bonding. Consequently, one may conclude that all the noncovalent interactions are driven by electrostatics. In fact, some authors do claim that everything *is* electrostatics (Politzer et al., 2015) and the rest is “*noumenon*”—the Kantian term introduced by Kozuch and Martin to describe unobservable explanations (Kozuch and Martin, 2013).

This simplified understanding does not explain why these noncovalent bonds are rigid. Stone in a recent paper entitled “Are halogen bonds electrostatically driven?” shows that for one selected Lewis acid, ClF, with a variety of bases, the directionality is determined by the exchange repulsion (Stone, 2013). Arguably, the first notice that the weak interactions with halogens were unusually rigid, i.e., have unusually high bending constants, was that by Klemperer and coworkers (Harris et al., 1974) in Ar-ClF. The first explanation of this phenomenon by Sadlej et al. (1993) centered on the pronounced dip in the exchange repulsion in the linear geometry that determines the shape of the potential curve. The dip in the repulsive term, as the work argues, allows the subsystems to approach more closely and bind stronger by induction and dispersion attractive components (no need for electrostatic component!). The dip in the exchange repulsion implies a depletion of the electron density in this position. The electron density depletion may also manifest itself as positive value of the electrostatic potential at the density map in this region. At this point, one may ask oneself what is the more sensitive and foolproof measure of the shape of electron density and the Lewis acid capability: the electrostatic potential, i.e., the interaction of a proton (positive point charge) with the charge distribution of a molecule, or the first-order exchange repulsion between the same charge distribution and a probe—such as, for example, a two-electron atom—the simplest Lewis base?

There exists an entire cottage industry around the analysis of the density shapes in terms of its gradient and Laplacian (Bader, 1994). This analysis is best suited for the detection of bond covalency and is not going to be employed here. The present work will focus on the role of Pauli exchange repulsion in probing the shapes of density in the sites of Lewis acidity and its consequences to the directionality of weak donor–acceptor interactions.

## Methods

The interaction energies between probing systems and Lewis acids are described in terms of the Symmetry Adapted Perturbation Theory (SAPT) (Jeziorski et al., 1994). The interaction energy is defined as a sum of electrostatics (es), exchange (exch), induction (ind), exchange-induction (ex-ind), dispersion (disp), and its exchange counterpart (ex-disp),

$$E_{SAPT0}=E_{es}^{\left(10\right)}+E_{exch}^{\left(10\right)}+E_{ind,r}^{\left(20\right)}+E_{ex-ind,r}^{\left(20\right)}+E_{disp}^{\left(20\right)}+E_{ex-disp}^{\left(20\right)}+\delta_{HF}$$ (1a)

$$E_{SAPT2+}=E_{SAPT0}+E_{es,r}^{\left(12\right)}+E_{exch}^{\left(11\right)}+E_{exch}^{\left(12\right)}+E_{ind}^{\left(22\right)}+E_{ex-ind}^{\left(22\right)}+E_{disp}^{\left(21\right)}+E_{disp}^{\left(22\right)}$$ (1b)

where “r” in the subscripts refers to the response-inclusive treatment of induction and electrostatics. The numbers in the superscripts in the $E^{\left(VW\right)}$ notation correspond to the orders in the intermolecular perturbation operator V and the intramonomer correlation operator W. The last term $\delta_{HF}$ stands for higher-order induction and/or residual effects. Two levels of SAPT are employed: SAPT0 means that the interaction energy terms are derived from the product of Hartree-Fock (HF) subsystem wave functions. We use terminology employed in the Psi4 program (Parrish et al., 2017; Hohenstein et al., 2011). In SAPT0, the terms on the right in Eq. 1a sum up to the HF + Dispersion level of theory. A higher level of theory is obtained if the monomer wave functions are correlated to the 2nd order in the intramonomer correlation operator W. This results in SAPT2. The level designated SAPT2+ (Parker et al., 2014) means that the treatment of the dispersion energy is enhanced up to the inclusion of triples (although the exchange counterpart of dispersion is not correlated). In the graphs below when SAPT2+ is employed, we fold the intramonomer correlation contributions into the respective terms and combine the induction with exchange (denoting it E_ind-PT_), as well as the dispersion with its exchange counterpart. The residual term $\delta_{HF}$ is shown separately for the reasons described below. The monomer geometries were taken from experiment (Huber et al., 2022) and the intersystem distances were either at the sum of van der Waals radii or at their equilibrium, if known (Evans et al., 2010; Hapka et al., 2013). The SAPT computations were performed using the Psi4 (Parrish et al., 2017) program suite, which employs the density fitting and Laplace transform for the evaluation of energy components (Hohenstein et al., 2011). The basis set was primarily aug-cc-pVQZ (Pritchard et al., 2019) except for the heavier atoms, I and Au, for which the def2-QZVPPD basis set was used (Pritchard et al., 2019) (this choice was dictated by the existence of density fitting basis sets in SAPT).

## Results and Discussion

### Convex, Concave, or Polar-Flattened?

**FIGURE 1:**
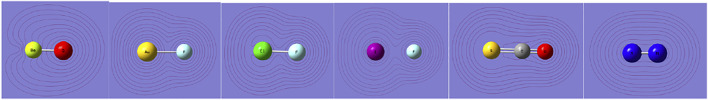
Density contours (0.001 au) of BeO, AuF, Cl-F, I-F, SCO, and N_2_ molecules from TPSS/AVQZ-derived densities.

We begin with a look at the shapes of electron density—a simple but crude instrument. We focus on density around a Lewis acidic site. Figure 1 shows the isodensity plots for a number of molecules discussed in this work. Going from the left to right, the density contours show the deep depletion in BeO, still visible depletion in AuF, a slight depletion in ClF, especially in a short region of Cl, an axial flattening of density in I-F around the iodine, convex contours in SCO around sulfur with some axial flattening, and finally the convex contours in N_2_ with no flattening. The axial flattening, also called polar flattening in literature (Sedlak et al., 2015), does not occur in N_2_; rather, its density protrudes axially in the lone pair region. In fact, the quadrupole moment of N_2_ is negative, thus indicating that the negative charge extends further along the molecular axis.

Now, how is this picture reflected in the electrostatic potential mapped on the threshold density?

**FIGURE 2:**
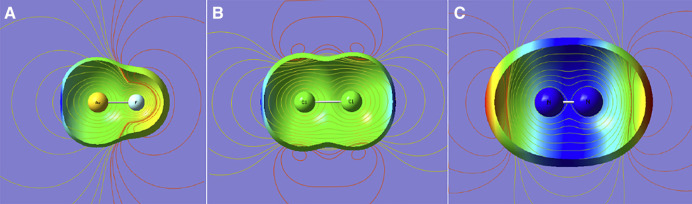
Density (0.001 au), electrostatic potential mapped on the density surface (blue for positive and red for negative), and electrostatic potential contours (yellow for positive; red for negative). From **(A–C)**: polar molecule (AuF), nonpolar molecule with positive quadrupole (Cl_2_), and with negative quadrupole (N_2_).

Three limited cases are presented in Figure 2: a dipolar and two quadrupolar cases. It shows the contours of the electrostatic potential that emanate from a dipole and two quadrupoles of opposite signs. One may conclude that the positive “σ hole” in the left two and the negative “σ bump” offer no unique insights except for pointing to the sign of the leading multipole moment of the molecule. Arguably, when the density surface shows an axial indentation, as in AuF in Figure 2, the magnitude of electrostatic potential at this point is enhanced. Nevertheless, the insights from this picture are qualitative at best.

Recent years have seen an explosive growth (Web of Science Core Collection, 2022) of literature on the features of electrostatic potential painted on the density (Cavallo et al., 2016; Kolář and Hobza, 2016). The types of holes that emerged from these studies range from σ to π to δ (Angarov and Kozuch, 2018). Simultaneously, the names of bonds associated with these “holes” exploded up to 8 by the last count (Zierkiewicz et al., 2021). The “hole” properties such as the spatial extent of the positive region (Kolář et al., 2015), the value of the maximal electrostatic potential, V_smax_, at a certain threshold density (Politzer et al., 2013), and the extent of the hole’s “polar flattening” (Sedlak et al., 2015) have been correlated with the bonding strength of a complex.Simultaneously, there were claims that only the density and its associated electrostatics represent “phenomena” and all the other computable and physically interpretable effects represent “unicorns” (Politzer et al., 2015). This literature avalanche gives an impression that all of the donor–acceptor interactions are electrostatically controlled, which is incomplete if not misleading. There are some dissenting voices from this chorus stressing the importance of the exchange effect (Adhikari and Scheiner, 2012; Stone, 2013), the ground-state charge-transfer interactions (Wang et al., 2014; Rezac and de la Lande, 2017; Thirman et al., 2018), orbital interactions (Pascoe et al., 2017), or covalency contributions (Bora et al., 2017).

The electrostatic effect and “σ holes”, which are expected to explain the directionality of these interactions, are purely classical concepts. To fully deconstruct the properties of the “hole” and to detect the existence of density depletions in the direction of the “hole”, we need a more sophisticated tool—a quantum effect of exchange. This requires the participation of at least a two-electron interacting partner.

### He-Molecule Interactions

**FIGURE 3:**
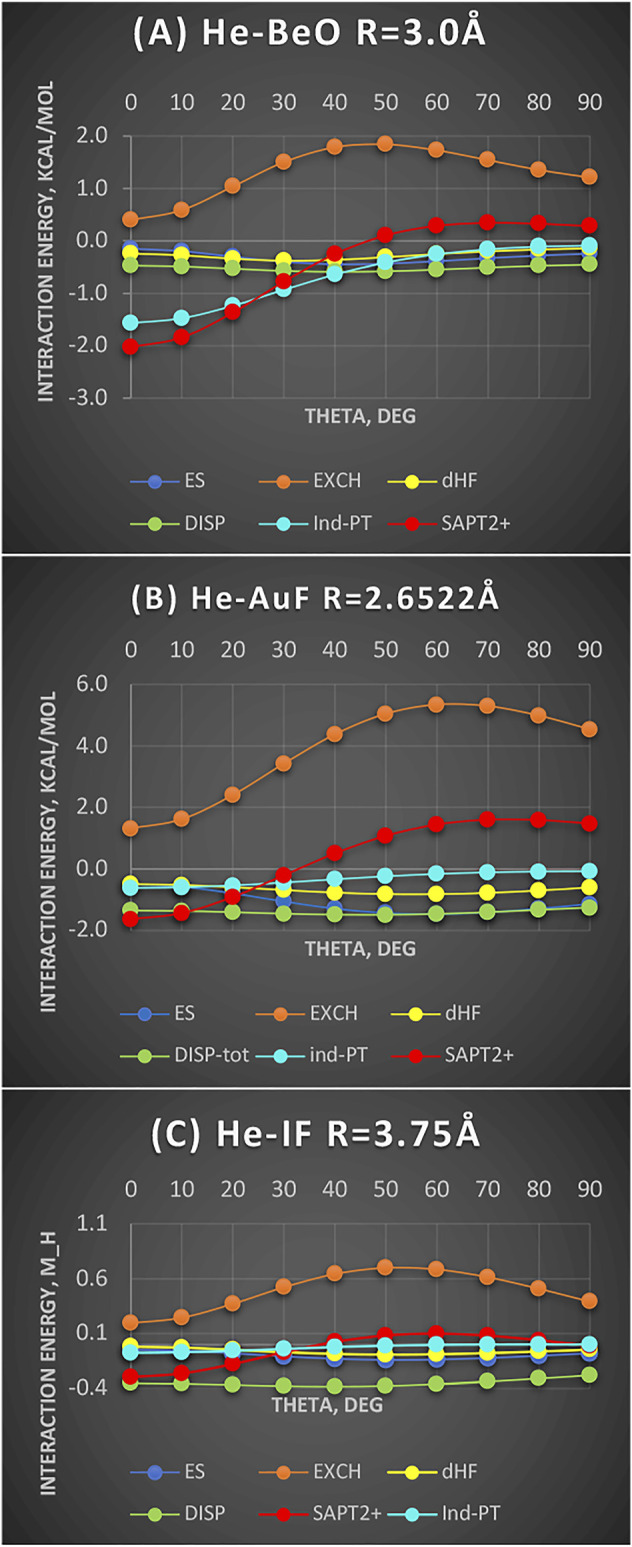
Interaction energy terms for angular displacement in Jacobi coordinates: **(A)** He-BeO R = 3.0 Å (units kcal/mol), **(B)** He-AuF R = 2.6522Å (units kcal/mol), and **(C)** He-IF R = 3.75 Å (units mH).

Figure 3 shows three complexes, He-BeO, He-AuF, and He-IF. In the first complex, the density hole is evident and so deep that it can accommodate a He atom but not Ne. In the second one, the density still has some depletion but to a much lesser degree. In the third one, only a flattening is seen (Figure 1). We will show that regardless of the depth of the density shape, the same pattern persists if the energy components are considered. The systems are also chosen by steeply decreasing dipole moments from 6.6D (BeO) to 4.5D (AuF) to 2.7D (IF) (as per CCSD computation). Despite these widely varying electrical properties, the nature of bending potentials remains the same. It is the exchange repulsion term that displays a deep “hole” for the linear configuration and thus dictates the shape of the overall interaction energy (shown as SAPT2+ in Figure 3). The electrostatic term favors nonlinear geometries and disfavors the linear ones. It is of no importance since it is of the overlap character. The induction term’s importance reflects the size of the dipole moment: the effect is maximized in He-BeO and minimized in He-IF. The dispersion energy is an important attractive contributor but its angular variations are minimal.

The same behavior could be expected for the radial coordinate: Namely, the depletion of the exchange repulsion allows the subsystems to approach more closely and benefit from enhanced dispersion and induction effects.

The *cavity in the interaction potential* of He-BeO was first demonstrated in the work of Hapka et al. (2013) in the form of zero-interaction energy boundary showing a deep indentation for the linear geometry on the Be side. They estimated the binding energy of nearly 1,900 cm^−1^ or 5.4 kcal/mol. Ne is similarly bound to BeO but penetrates less into the cavity by 0.267 Å according to Nunzi et al. (2017). The Ar-BeO study estimated the well depth of 3,990 cm^−1^ or 11.4 kcal/mol (Tebai et al., 2014), quite unusual for the rare gas binding to a molecule. The SAPT conclusion is that the minimum in He-BeO is largely due to the exchange depletion reinforced by the induction effect. The work of (Nunzi et al., 2017) goes even further by postulating that the unique strength of He-BeO as compared to Ne-BeO, for example, is due to the ability of BeO to back-donate to the vacant 2p orbitals of He—and the inability to do so to Ne because its 2p orbitals are already occupied. Our observation is that (1) Ne is simply too large to penetrate as deep into the density depletion in BeO as He could and (2) the induction effect is actually stronger in Ne than in He.

**FIGURE 4:**
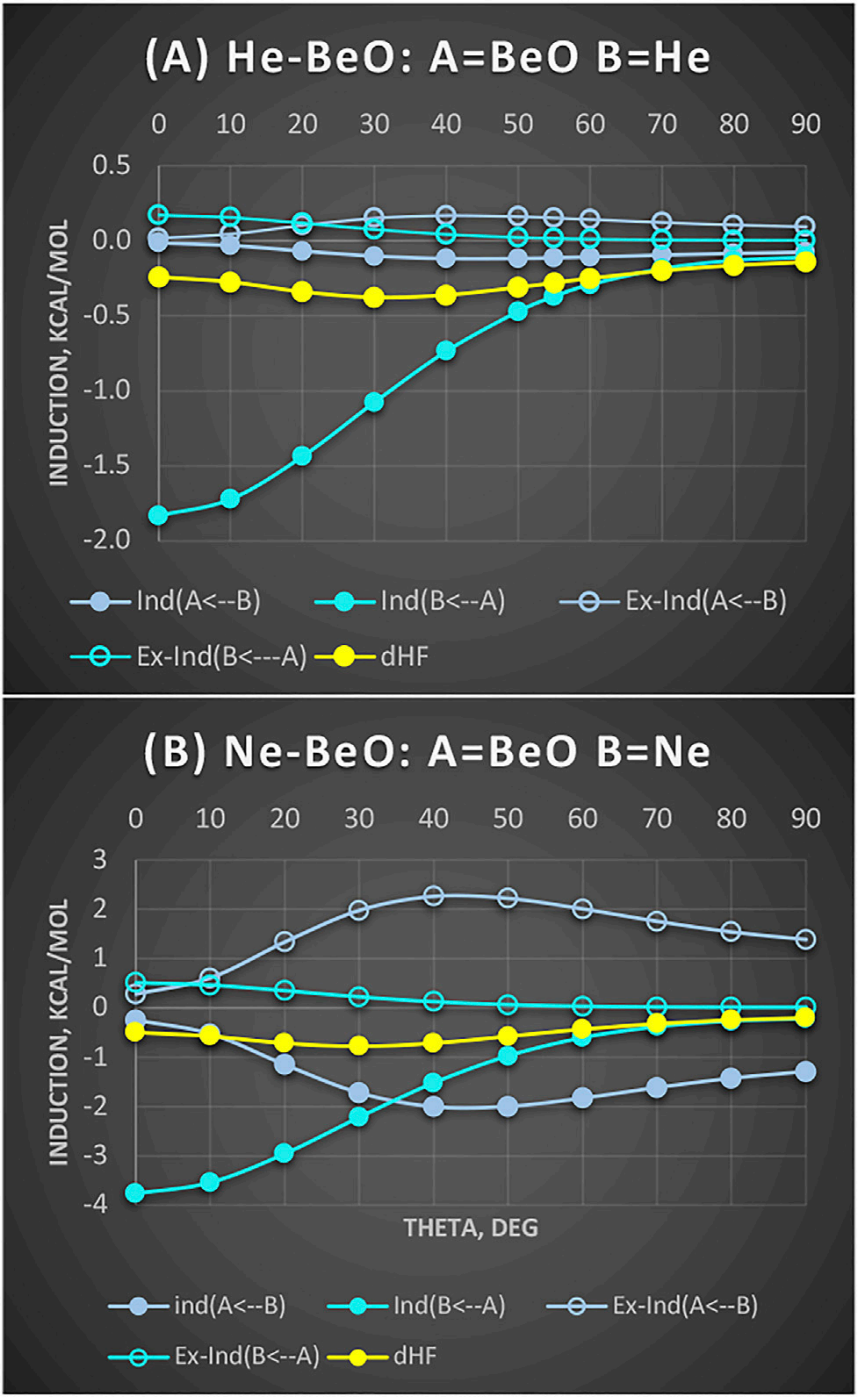
He-BeO **(A)** and Ne-BeO **(B)** at R = 3.0 Å: Partitioning of induction terms into mutual contributions of A = BeO and B=He/Ne. Solid circles are induction terms and open circles are exchange-induction terms. Values in kcal/mol.

We examine this intriguing hypothesis of back-transfer below (Figure 4). First, one expects that if there is an overlap between the occupied π MOs of BeO with vacant 2p orbitals of He, it should favor the linear configurations. Second, it should manifest itself in the Molecule → Atom induction contribution. These contributions (along with a residual δ_HF_) are shown in Figure 4 for both He and Ne complexes with BeO taken at the same distance R = 3.0 Å, which is the equilibrium for Ne-BeO.

It appears that BeO → He/Ne contributions are strongly attractive, favoring the linear structure. Their respective exchange counterparts cancel very small fractions of these attractions.

The magnitudes of the induction terms of the BeO → He/Ne type are consistent with ca. 2:1 ratio of static dipole polarizabilities of Ne to He. In both cases, the BeO → He/Ne induction attraction strongly favors the linear structure. What if the donation to virtual 2p orbitals shows up in the residual term δ_HF_? Here, in neither case is δ_HF_ the most attractive for the linear geometries. Thus, in the SAPT language, the only indication to support the BeO-to-He donation is in very strong BeO → He induction. One observation, however, is worth emphasizing: Namely, in the Ne-BeO complex, we can see very strong, common sense eluding Ne → BeO contribution of ca. 2 kcal/mol at skewed geometries. Thankfully, the exchange counterpart of this term varies as almost its mirror image and nearly completely wipes out the common-sense-defying effect. It is useful to interdict to explain why we use the term “common-sense-defying.” This is because the induction effect allows electrons of one monomer to occupy the already occupied orbitals of the partner, thus violating the Pauli principle, if unconstrained by the exchange. The lesson from this is that employing the induction effect alone is error prone. The induction energy must be accompanied by its exchange counterpart, if unphysical effects are to be avoided.

### When is the Electrostatic Effect Important?

To determine the effect of the nonsphericity of halogen on the shape of the interaction potential, we examine a series of electron donors (Lewis bases) with one Lewis acid ClF, the latter showing the prominent “σ hole,” to establish in what circumstances is electrostatic effect the controlling one. This question was previously explored in the literature (Stone, 2013), but we intend to place it in a wider context.

**FIGURE 5:**
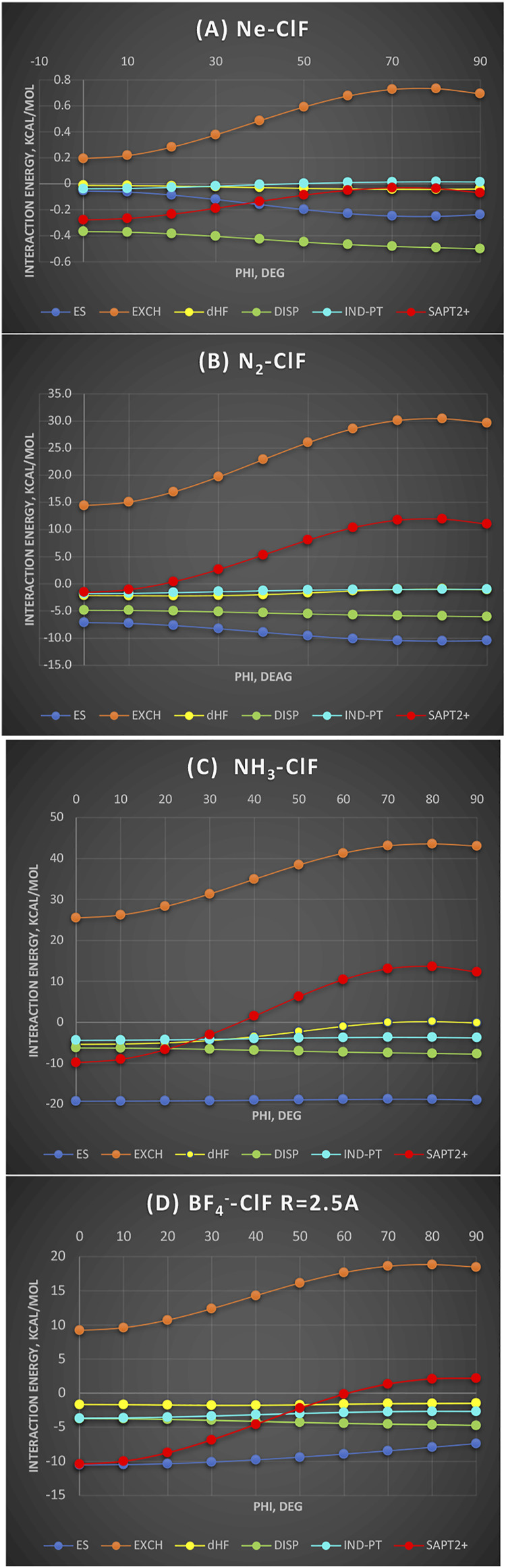
SAPT energy components as a function of ClF bending around the Cl atom (denoted phi); values in kcal/mol. **(A)** Ne-ClF at the sum of van der Waals radii distance 3.3 Å; **(B)** N_2_-ClF at the N-Cl distance 2.5 Å; **(C)** NH_3_-ClF at the N-Cl distance 2.5 Å; **(D)** BF_4_^−^-ClF at the intersystem F^−^-Cl distance 2.5 Å.

In Figure 5, four Lewis bases are shown interacting with ClF, from very weak to ionic, as functions of the bending angle of ClF around the Cl atom (denoted φ) from linear to perpendicular. It is noteworthy that in neither case is the induction effect controlling the shape of the bending potential and neither is the dispersion energy nor the electrostatic. The Ne-ClF complex’s (Figure 5A) total SAPT2+ interaction energy is the most binding for the linear geometry owing to the exchange repulsion’s minimum of ca. 0.5 kcal/mol compared to the perpendicular arrangement. Indeed, this is the true manifestation of the hole. The electrostatic effect is overlap-dependent and varies in the opposite way. Two σ donors described in what follows, N_2_ and NH_3_, are considered at the same intersystem distance N---Cl of 2.5 Å, which equals the equilibrium separation in the latter. In the BF_4_^−^-ClF complex, the same intersystem distance 2.5 Å is employed because it is close to the complex’s equilibrium separation. In a non-polar N_2_ interacting with ClF, the exchange hole amounts to 15 kcal/mol, dominating the total SAPT2+ energy. In fact, one curve appears to be the vertical translation of the other. The only other term that favors the linear geometry is δ_HF_ (see below for more discussion). In the polar NH_3_ interacting with ClF, considered at the same intersystem distance, the exchange hole amounts now to 18 kcal/mol. The exchange effect again controls the shape of the bending potential as well as the well depth of this complex. Surprisingly, given the participation of the two polar subsystems, the electrostatic energy is almost constant with angular variation between −19.2 kcal/mol at 0° and −19.0 kcal/mol at 90°. Similarly, the induction effect (more specifically its perturbation theory part, E_Ind-PT_) is also nearly constant (to 0.6 kcal/mol). To emphasize: the electrostatic and induction effects are *isotropic*, or nearly so, around the Cl end of the molecule. This appears to contradict the electrostatic predominance due to “σ hole” and, in particular, attributing any predictive capabilities to the value of V_smax_. The second most anisotropic term is the residual δ_HF_ contributions to which we will return below.

Finally, in the ion-dipole complex BF_4_^−^-ClF, one would expect that the electrostatic energy should drive the interaction. However, it is still the exchange hole of over 9 kcal/mol that shapes the potential curve. The electrostatic energy varies only by ca. 3 kcal/mol between 0° and 90°. All the other terms remain constant or nearly so (*cf*. dispersion). Naturally, at longer separations, one would expect the electrostatics to dominate; this is because the electrostatic effect varies more slowly with the intersystem distance than the exchange effect.

To summarize, with the variety of electron donors, ranging from a rare gas to quadrupolar to dipolar to ionic interacting with ClF, the bending potential around the equilibrium separation is controlled by the exchange hole. The effects of the electrostatic “σ hole” of ClF manifesting themselves in the electrostatic and induction interactions are only secondary.

### Where Is the Charge-Transfer Contribution?

The complexes considered in the section *When is the Electrostatic Effect Important?* above were halogen-bonded, which are believed to be stabilized also by the charge-transfer contribution, as predicted by the Mulliken’s interpretation of the complexes of this type (Mulliken and Person, 1969) (before they became known as halogen-bonded); although the subject remains controversial. There have been several schools of thought on inclusion of charge-transfer energies in the interaction energies beginning with Morokuma partitioning of the HF interaction energy in the early 70s (Morokuma, 1971; Kitaura and Morokuma, 1976), the Stone–Misquitta monomer-to-dimer basis set extension of the induction effect in SAPT (Stone and Misquitta, 2009) [see also recommendation in Psi4 (Parrish et al., 2017)], Misquitta’s attempt to “regularize” one-electron V operator terms (Misquitta, 2013), constraining intermonomer DFT and then relaxing it (Wu and Van Voorhis, 2005; Lao and Herbert, 2016), and the energy decomposition scheme ALMO-EDA in its latest variant (Thirman and Head-Gordon, 2015), just to name a few. Finally, there are also views that the charge transfer is fully included in the induction effect (Politzer et al., 2013; Sedlak et al., 2015).

In the halogen bond context, the charge-transfer contribution is understood as energy lowering resulting from the orbital interaction involving the lone pair of Lewis base (n) with the LUMO of halogen molecule $\sigma^{*}$ of the type (Thirman et al., 2018)

$$E_{CT}=\frac{\langle n\left|\hat{H}\right|\sigma^{*}\rangle }{E_{n}-E_{\sigma^{*}}}$$ (2)

This interaction favors the direction where n and $\sigma^{*}$ overlap best, i.e., the linear geometry. It also benefits from the denominator’s energy rising of n and lowering $\sigma^{*}$ resulting from the interaction. Since perturbation theory is based on the product of unperturbed monomers’ wave functions, the latter effect will be absent. If one performs the full dimer HF energy calculations, the orbitals that are still located largely on the respective monomers will be relaxed. Therefore, the term $\delta_{HF}$ , which is obtained as

$$\delta_{HF}=\;\text{Δ}E_{HF}-E_{es}^{\left(10\right)}-E_{exch}^{\left(10\right)}-E_{ind,r}^{\left(20\right)}-E_{ex-ind,r}^{\left(20\right)}$$ (3)

where $\Delta E_{HF}$ stands for the supermolecular interaction energy at the HF level, should capture such effects. A conventional view of $\delta_{HF}$ is that it sums up induction and exchange-induction terms of orders higher than the second. However, it also includes other residual effects including a possible divergence of SAPT due to the weak antisymmetry forcing.

In Figure 5, we purposely showed perturbation-theory-derived induction effects, E_ind-PT_, as separate from $\delta_{HF}$ to test the hypothesis of $\delta_{HF}$ including the charge-transfer effects.

In Ne-ClF, $\delta_{HF}$ (Figure 5A) is near zero for the linear geometry and more attractive for the bent geometries. Upon closer inspection, we may notice that in the bent geometries, E_ind-PT_ becomes positive—an unphysical behavior. Thus, $\delta_{HF}$ in this case corrects the SAPT’s convergence. In BF_4_^−^-ClF (Figure 5D), $\delta_{HF}$ is constant to a fraction of kcal/mol while E_ind-PT_ varies by 1 kcal/mol in favor of the linear geometry. Therefore, $\delta_{HF}$ is expected to be of induction origin. We should contrast these two cases with what happens in NH_3_-ClF. In this instance, when we sum up all the perturbational SAPT terms, the well depth is too shallow by half and $\delta_{HF}$ provides the missing half. $\delta_{HF}$ is strongly attractive for the linear structure and vanishes for the perpendicular one. This is the typical dependence that one would expect from the charge-transfer effect from n to σ*. In N_2_-ClF, considered at the same distance, $\delta_{HF}$ binding exceeds that from E_ind-PT_ and again is the most attractive at the linear geometry. However, it does not vanish at the perpendicular geometry indicating the presence of some residual effects in strongly repulsive part of the interaction SAPT2+ potential.

To conclude, $\delta_{HF}$ may contain, depending on the type of a complex, a variety of effects: residual induction and exchange in a weak one with a rare gas, Ne-ClF, or strong but with similarly nearly spherical donor BF_4_^−^-ClF. In the paradigm “charge-transfer”, halogen-bonded complex, NH_3_-ClF, one-half of the binding energy in the minimum resides in $\delta_{HF}$ , i.e., the term whose angular dependence is consistent with the orbital interaction between n and σ*. Thus, we posit that when charge-transfer energy contributions are warranted (e.g., by the right symmetry and energetical balance of donor–acceptor properties), they would manifest themselves in SAPT in its residual term $\delta_{HF}$ .

Finally, we should mention that the use $\delta_{HF}$ is only compatible with SAPT based on HF subsystem zero-order wave functions because only in this case, as proved by Moszynski et al. (1996), do the induction terms summed up to the infinite order in V converge to the HF interaction energy (sans the first-order energy). As observed before (Modrzejewski et al., 2012), if SAPT is based on the DFT monomer description, the inclusion of $\delta_{HF}$ , which is common practice in the literature to improve results, is problematic.

### A New Bond Type or the Exchange Hole?

**FIGURE 6:**
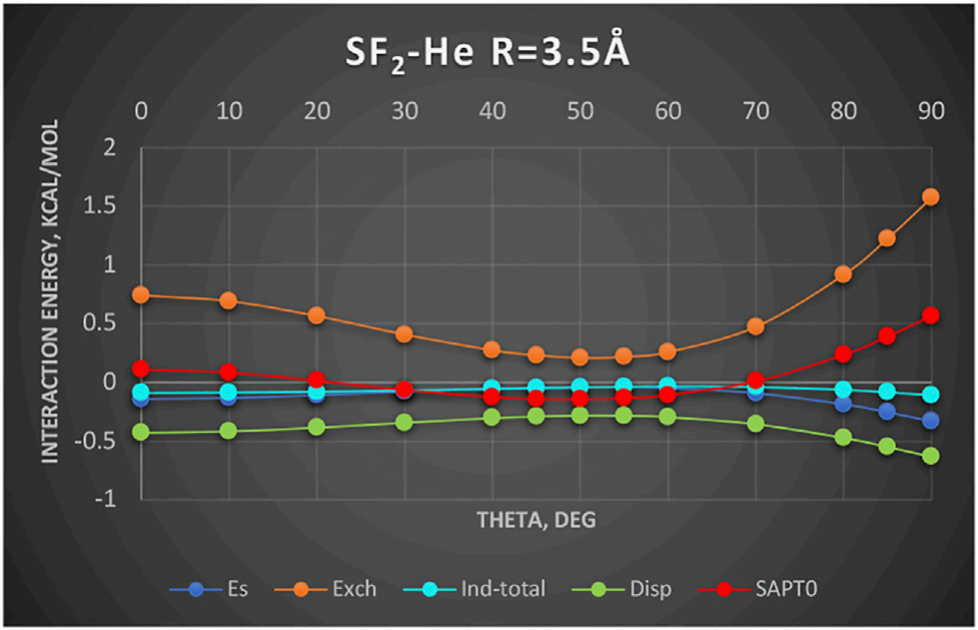
SAPT0 energy components of SF_2_-He as a function of theta. Complex is described in Jacobi coordinates: R = 3.5 Å and theta = 0° corresponds to He located at the C_2_ axis on the S side. Values in kcal/mol.

**FIGURE 7:**
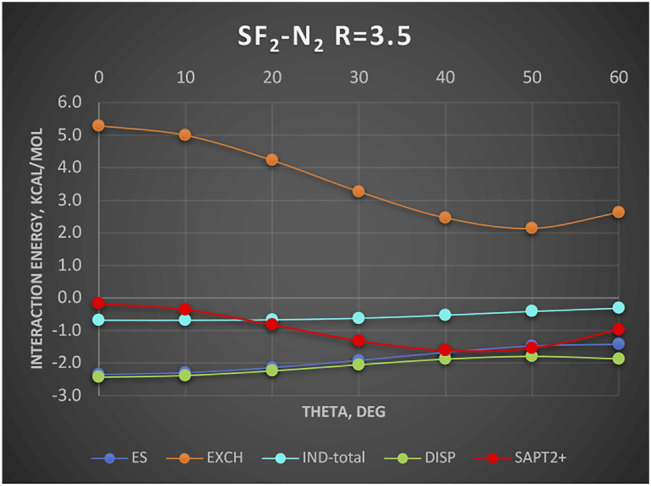
SAPT2+ energy components of SF_2_-N_2_ as a function of theta. Complex is described in Jacobi coordinates: R = 3.5 Å and theta = 0° corresponds to N_2_ oriented at the C_2_ axis on the S side. Values in kcal/mol.

A new and unique bond has been attributed to the 16-group containing atoms O, S, Se, etc., the chalcogen bond (Pascoe et al., 2017; Wang et al., 2009). In this section, we examine an example chalcogen-bond donor SF_2_. The electrostatic potential of SF_2_ as shown in Angarov and Kozuch (2018) features two positive regions on both sides of the C_2_ axis roughly perpendicular to the S–F bonds. These positive regions are supposed to attract basic regions of a Lewis base. To determine what is really behind this attraction, we show in Figure 6 the SAPT results for the complex SF_2_-He. The Jacobi coordinate R = 3.5Å corresponds to the sum of van der Waals radii of He and S and also to the radial minimum in the SAPT2+ potential surface. The angular potential (here derived at the SAPT0 level of theory) is shallow with a weak minimum at theta = 50°. The minimum closely follows the angular variation of the exchange repulsion. Both the electrostatic and induction effects contribute minimally, especially in the minimum region. The only attractive term of some importance is the dispersion energy albeit the least attractive around the theta = 50° angle. Our two-electron probe thus indicates that the controlling effect is the exchange depletion—an exchange hole. The electrostatics and induction are unimportant. Would the same principles apply in the case of nonspherical Lewis base? The work of Angarov and Kozuch (2018) shows that when the Lewis base is a σ-donor HCN molecule, the angle of approach is also tilted ca. 53° away from the C_2_ axis just as in the SF_2_-He discussed above. Their results show only the total interaction. In a quick look at the actual balance of the energy components, we show in Figure 7 the interaction with another σ-donor N_2_: N_2_-SF_2_. The SAPT2+ curve again follows closely the exchange depletion at the tilted geometry with theta = 50°, just as in SF_2_-He. The electrostatic energy favors the axial geometry (theta = 0°) and only coincidentally matches the value of total interaction energy around the minimum. The dispersion energy shows a similar pattern to SF_2_-He: the most attractive at theta = 0 and the least attractive in the tilted geometry.

What these two cases have in common is that the Lewis base approaches the C_2_ axis of SF_2_ at a tilted angle and this happens regardless of the polarity state of the base. This may create an impression that the base is electrostatically attracted to the “σ hole” on the side of the sulfur atom (or, more accurately, above the S–F bond). In reality, this position is preferable due to the depletion in the exchange repulsion. Could this be viewed as a unique chalcogen bond? To answer this question let us recall a 30-year-old perturbation theory result of another chalcogen: H_2_O-Ar (Chałasiński et al., 1991). In this complex, Ar is also tilted with respect to the C_2_ axis of H_2_O, the fact that was confirmed experimentally by the high-resolution vibration–rotation–tunneling spectroscopy (Cohen and Saykally, 1993). The perturbation-theory paper (Chalasinski et al., 1991) states that the angle is determined by the depletion in the exchange repulsion. Incidentally, the electrostatic potential of H_2_O does not feature a sigma hole in this region. The σ holes, if we can call them such, reside on H atoms!

### The Roots of the Exchange Holes

**FIGURE 8:**
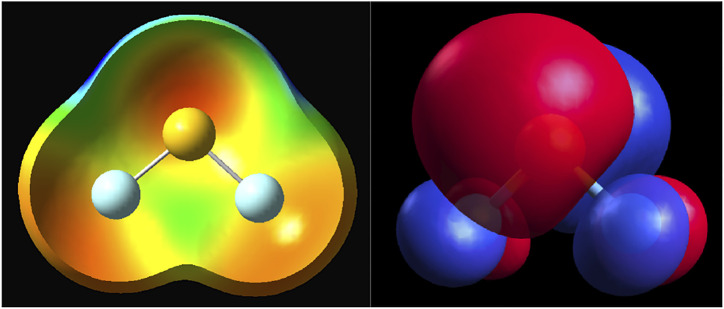
SF_2_: The electrostatic potential mapped on the density surface of 0.001 au threshold (left); HOMO orbital (right) from DFT TPSS calculations.

The final question concerns the cause of the exchange depletion in the systems studied here and in many other cases discussed in the vast literature of novel non-covalent bonds. The electron density depletion shown in Figure 1 may be obvious in some molecules, such as BeO, but even in the halogen bond donors, the density depletions are not obvious from the density contours. The first-order exchange repulsion detects regions that are, to a varying degree, Pauli-forbidden and thus serve as a very sensitive probe of the doubly occupied space. The regions that are not occupied invariably involve nodes and nodal planes. It is, for example, axiomatic and taught in undergraduate courses that the nodal planes help distinguish bonding from antibonding orbitals or that the T_2g_ symmetry positions in the octahedral symmetry are more ligand-favorable than E_g_ because they lie on the intersections on the d_π_ orbitals of the transition metal. The same principle applies to the exchange holes of the systems studied here (and elsewhere). A good example is the case of SF_2_ shown in Figure 8. On the left, there is the electrostatic potential mapped on the density, and on the right, the highest occupied molecular orbital (HOMO) of the molecule. The depletions seen above in the exchange repulsion (Figure 7) occur on the intersection of the nodal plane of the π orbital in plane of the molecule and another node that runs perpendicular to the S–F bond.

All Lewis acids examined in this work have the same features: the regions of depleted density occur on the intersections of nodal planes (the illustrations are included in the Supplementary Material). One easily explainable exception to this rule among the present systems involves AuF (Supplementary Figure S2). In AuF, as seen in Figure 3, the exchange depletion occurs in the axial position on the Au side whereas the HOMO orbital is d_σ_ along the molecular axis. However, due to the relativistic contraction of the 6s orbital in Au, the axially extending part of s + d_z2_ hybrid is minimized. Close-lying d_π_ and d_δ_ occupied orbitals all feature one and two nodal planes, respectively, on the Au side of the molecule.

Molecular orbitals are, after all, one-electron functions and, to an extent, *noumena*. However, their symmetries have a deeper meaning as the irreducible representations of the nuclear point group. Our point here is that the depletion of the density on nodal planes has consequences to bonding whether covalent or noncovalent, indeed.

The circumstances of an actual electron vacancy as in a Cl radical, often mentioned as a protagonist of the halogen bond (Politzer et al., 2010; Kolář and Hobza, 2016), are entirely different. A ^2^P state of Cl is triply degenerate and it takes an interaction with another system to remove this degeneracy. The Pauli repulsion along the singly occupied orbital with a closed-shell system will be smaller than along the doubly occupied one giving rise to multiple, coupled potential energy surfaces (Klos et al., 2004). If that system is a positive point charge, as in the case of the electrostatic potential, the interaction in the direction of singly occupied orbital will also be more repulsive as shown in (Politzer et al., 2010) for electrostatic reasons. The open-shell case brings us back to the original meaning of the electronic hole (Koch et al., 1987), which, by and large, is not governed by the nodal surfaces but by an actual electron vacancy. A transplantation of this concept to the closed-shell case of halogen bonding has been a gross simplification.

## Conclusion

We investigated a number of prototypical weak electron donor–electron acceptor complexes by the Symmetry Adapted Perturbation Theory, some of which belong to novel classes of weak bonds such as halogen and chalcogen bonds. Also included were weak complexes involving “unnamed as yet bonds” of electron acceptors such as BeO and AuF. The recent literature trend is to associate these novel bonds with a variety of “holes”, σ, π, δ, or positive areas in their electrostatic potential maps. The presumption is that these positive areas of the electrostatic potential are indicative of the electrostatic nature of these noncovalent bonds. This naïve electrostatic view extends to the explanations of the directionality of approaches between the subsystems forming these bonds. We show that one common feature of these electrostatic potential “holes” is the local depletion of electron density of which the best detector is the first-order exchange repulsion. The minimization of this repulsion determines the bond directionality and its relative angular rigidity. In relatively strong complexes of BeO with rare gases, where BeO shows a clear cavity in electron density—an ultimate “σ hole”—the electrostatic effect *is not the controlling* component—the exchange repulsion is. In halogen bonds, the halogen atom is nonspherical with an axial “σ hole” in its electrostatic potential, but in no case examined from rare gas to polar to anionic is the electrostatic energy responsible for the directionality of the halogen bond. In fact, it is not even maximized in the direction of the σ hole in N_2_-ClF and NH_3_-ClF! Yet, in all the cases, the exchange repulsion is minimized in the direction of the σ hole. The IUPAC definition (Desiraju et al., 2013) states that “forces involving the formation of the halogen bond are mainly electrostatic”. There are some extra qualifiers to this statement adding polarization, charge transfer, and dispersion, but not a word about the exchange repulsion. It is hoped that the present work will shift this paradigm.

The charge-transfer energy components, which are somewhat controversial, even though the halogen bonds used to be called charge-transfer complexes, were examined here in the framework of SAPT. Although, by design, SAPT is not able to yield charge-transfer interaction as a well-defined energy term, despite some admirable attempts in literature (Misquitta, 2013; Stone and Misquitta, 2009), we postulate that if these effects are important, as in NH_3_-ClF, they would reside in δ_HF_—the residual term encompassing SAPT unaccounted for terms that occur in the well-defined supermolecule’s energy. It is also important to stress that δ_HF_ is only strictly compatible with the SAPT based on the HF zero-order wave functions.

In the paradigm chalcogen-bond donor SF_2_, the origins of the “hole” in the electrostatic potential [which, by some definitions (Angarov and Kozuch, 2018; Scheiner, 2021), would be called a π-hole] are exactly the same as in the σ hole case—the depletion of electron density on the intersection of nodal planes and consequent minimization of the exchange repulsion.

The minimized exchange repulsion associated with the subtle and less subtle depletions of the electron density occurs on the nodal planes or on the intersections of more than one nodal plane in the highest occupied molecular orbitals of a Lewis acid, provided that the systems are closed-shell. The role of nodal planes in covalent and coordinate covalent bonds is well recognized. This work points to their similarly equal importance in certain types of donor–acceptor noncovalent interactions, such as those studied in the present work.

## Data Availability

The original contributions presented in the study are included in the article/Supplementary Material, further inquiries can be directed to the corresponding authors.
