# Supplementary material for: Reassessing the Role of σ Holes in Noncovalent Interactions: It is Pauli Repulsion that Counts
Source: Front Chem. 2022 Apr 7;10:858946. doi: 10.3389/fchem.2022.858946 (PMC9021534; doi:10.3389/fchem.2022.858946)
Supplement: Supplementary file 1 [file DataSheet1.PDF]

***Supplementary Material***

to accompany

# Reassessing the role of σ holes in noncovalent interactions: It is Pauli repulsion that counts

by

Malgorzata M. Szczesniak and Grzegorz Chalasinski

1. Supplementary Figures

Molecular orbitals were obtained from DFT calculations with TPSS functional with aug-cc-pVQZ basis set (in the case of Au the basis set was aug-cc-pVQZ-PP with pseudopotential).

| | |
|---|---|
| 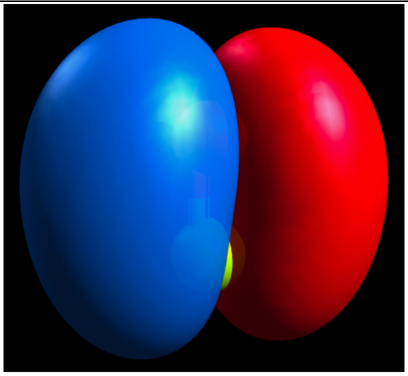 HOMO | 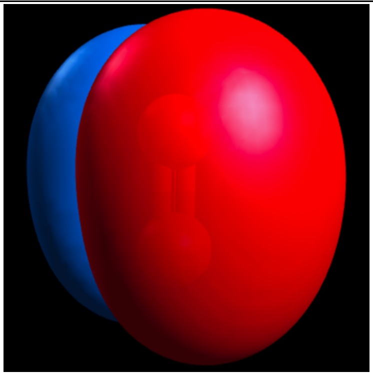 HOMO-1 |
| 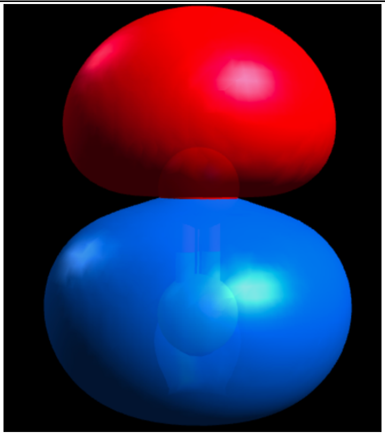 HOMO-2 | |

Figure S1: Highest occupied molecular orbitals of BeO; Be atom is on the top.

| | |
|---|---|
| 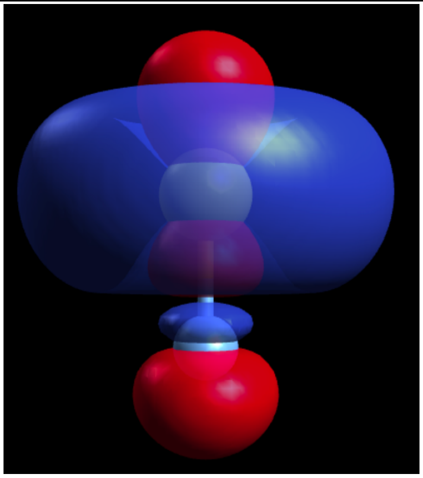 HOMO | 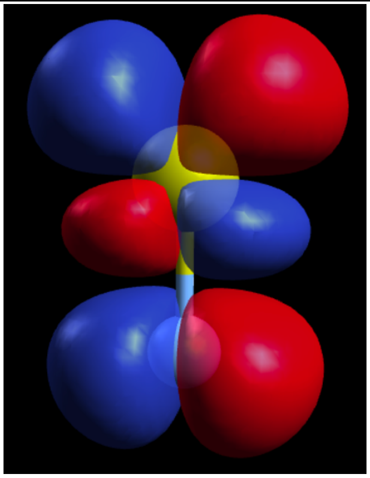 HOMO-1 |
| 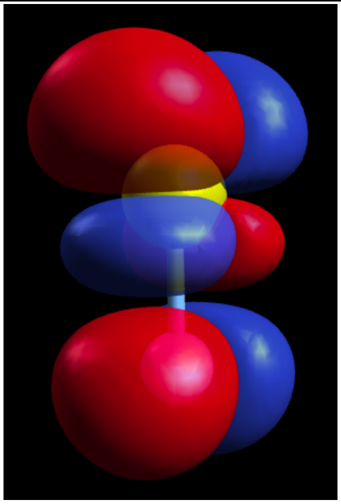 HOMO-2 | 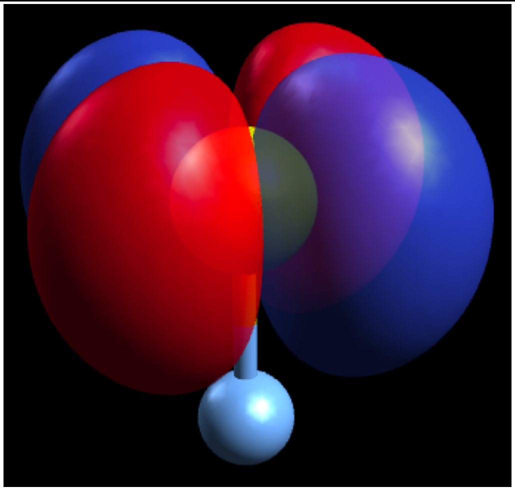 HOMO-3 |

Figure S2: Highest occupied molecular orbitals of AuF; Au is on the top.

| | |
|---|---|
| 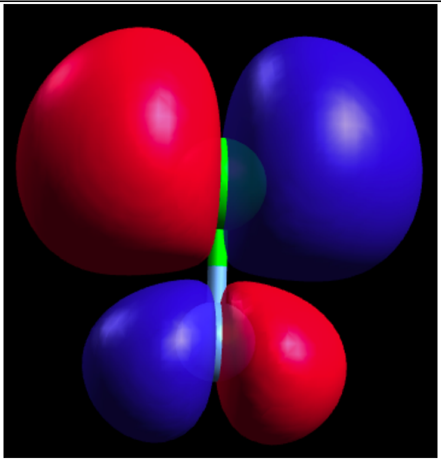 HOMO | 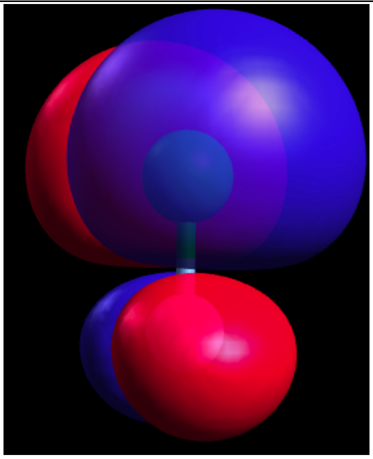 HOMO-1 |

| | |
|---|---|
| 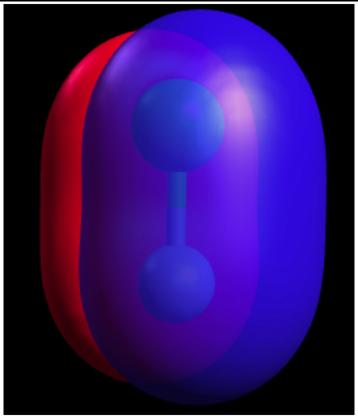 HOMO-2 | 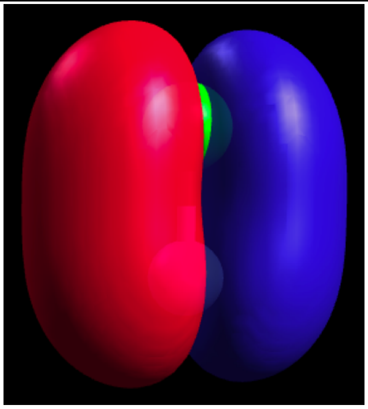 HOMO-3 |

Figure S3: Highest occupied molecular orbitals of ClF; Cl atom is on the top
